# Age-dependent decline in fin regenerative capacity in the short-lived fish *Nothobranchius furzeri*

**DOI:** 10.1111/acel.12367

**Published:** 2015-06-29

**Authors:** Sebastian Wendler, Nils Hartmann, Beate Hoppe, Christoph Englert

**Affiliations:** 1 Molecular Genetics Laboratory, Leibniz Institute for Age Research – Fritz Lipmann Institute (FLI)Beutenbergstr. 11, 07745, Jena, Germany; 2 Faculty of Biology and Pharmacy, Friedrich Schiller University of JenaFürstengraben 1, 07743, Jena, Germany

**Keywords:** aging, caudal fin, epimorphic regeneration, short lifespan, teleost killifish

## Abstract

The potential to regenerate declines with age in a wide range of organisms. A popular model system to study the mechanisms of regeneration is the fin of teleost fish, which has the ability to fully regrow upon amputation. Here, we used the short-lived killifish *Nothobranchius furzeri* to analyse the impact of aging on fin regeneration in more detail. We observed that young fish were able to nearly completely (98%) regenerate their amputated caudal fins within 4 weeks, whereas middle-aged fish reached 78%, old fish 57% and very old fish 46% of their original fin size. The difference in growth rate between young and old fish was already significant at 3 days post amputation (dpa) and increased with time. We therefore hypothesized that early events are crucial for the age-related differences in regenerative capacity. Indeed, we could observe a higher percentage of proliferating cells in early regenerating fin tissue of young fish compared with aged fish and larger fractions of apoptotic cells in aged fish. Furthermore, young fish showed peak upregulation of several genes involved in *fgf* and *wnt/*β*-catenin* signalling at an earlier time point than old fish. Our findings suggest that regenerative processes are initiated earlier and that regeneration overall is more efficient in younger fish.

## Introduction

Regeneration is commonly defined as the replacement of body parts lost by injury (Poss, 2010). The potential to regenerate is very differently distributed among animals and organ systems. Invertebrates such as *Hydra* and planarians have the potential to renew whole animals from small body parts, whereas many mammalian tissues and organs show strong limitations in their regenerative capacity. Nonmammalian vertebrates such as amphibians and fish are known for their remarkable capacity to regenerate significant parts of heart, spinal cord and limbs or fins. Already more than a century ago, it has been observed that partially amputated fins of teleost fish are capable of regeneration resulting in the complete restoration of epidermis, bones, blood vessels, nerves, connective tissue and pigmentation (reviewed in Iovine, 2007; Tal *et al*., 2010). Nowadays, epimorphic fin regeneration in fish has become an efficient model system because it is well accessible and the regenerative process can easily be followed.

The first step after fin amputation usually occurs within 24 h and includes that epithelial cells migrate over the wound and form the wound epidermis and apical epithelial cap. During the next stage, mesenchymal cells dedifferentiate and start proliferating as they migrate towards the apical epithelial cap. They form the so-called blastema which serves as a source of progenitor cells. Cells in the distal part of the blastema proliferate slowly and provide the direction and signals for the outgrowth, whereas cells in the proximal part of the blastema proliferate rapidly (Nechiporuk & Keating, 2002). These cells migrate further to appropriate locations and differentiate to populate the new tissue.

An important aspect in understanding regenerative processes is the knowledge about the cellular origin of the regenerated tissue. In general, cells of renewed tissues can be generated by two mechanisms, either involving stem or progenitor cells, or by dedifferentiation and/or transdifferentiation of existing cells (Poss, 2010). The main cellular origin during fin regeneration has been suggested to involve dedifferentiation of cells rather than multipotent stem cells. However, the question of cellular origin during fin regeneration is still a subject of debate (Tanaka & Reddien, 2011). Zebrafish fin regeneration has been also used to identify conserved gene functions during regeneration. Several signalling pathways including Fgf, Wnt, Tgf-β, Bmp and Hedgehog signalling have been described to be crucial for fin regeneration in zebrafish. Disruption of Fgf signalling, for example by inactivation of the *fibroblast growth factor receptor 1 (Fgfr1)* gene, blocks blastema formation and expression of the homeobox domain gene *msxb* which marks the cells in the distal part of the blastema (Akimenko *et al*., 1995; Lee *et al*., 2005; Thummel *et al*., 2006). Inhibition of Wnt/β-catenin signalling also prevents the early specification of the distal blastema (Stoick-Cooper *et al*., 2007; Chen *et al*., 2009; Wehner *et al*., 2014). Both pathways have been reported to be involved in all stages of regeneration from wound closure to regenerative outgrowth.

The regenerative capacity does not only differ among species and organs but also depends on developmental stage and age. In humans, for example, only children show an enhanced ability to regrow lost fingertips, whereas adults have lost this ability (Douglas, 1972; Illingworth, 1974). Moreover, young mice renew digit tips much more effectively than mature animals (Borgens, 1982; Reginelli *et al*., 1995). Thus, an interesting question is whether highly regenerative species such as fish keep their regenerative potential throughout their lifespan. To study possible age-related effects on the regenerative capacity, we used a short-lived teleost fish species, the turquoise killifish *Nothobranchius furzeri*. It has a maximum lifespan of 4–14 months depending on strain and origin, which is the shortest reported maximum lifespan of a vertebrate species in captivity (Valdesalici & Cellerino, 2003). This short lifespan has been considered to result from the unpredictable habitat as the fish live in ephemeral ponds in south-eastern Africa, an area of distinct dry and rainy seasons. During the dry season, the ponds can desiccate and the population survives as drought-tolerant embryos. When the ponds are filled with water, the embryos hatch, rapidly grow and reach sexual maturation after 3–5 weeks (Blazek *et al*., 2013). We and others have demonstrated that the short lifespan of *N. furzeri* is associated with rapid aging as shown by an early onset of aging biomarkers, a decline in learning and behavioural capabilities, age-related telomere shortening and an age-related impairment of mitochondrial function (Terzibasi *et al*., 2008; Hartmann *et al*., 2009, 2011).

In the present study, we addressed the questions whether different aspects of epimorphic fin regeneration are affected during aging in *N. furzeri*. We therefore performed a comprehensive study analysing outgrowth kinetics, number of proliferating and apoptotic cells, and gene expression at different ages.

## Results

**Fig 1:**
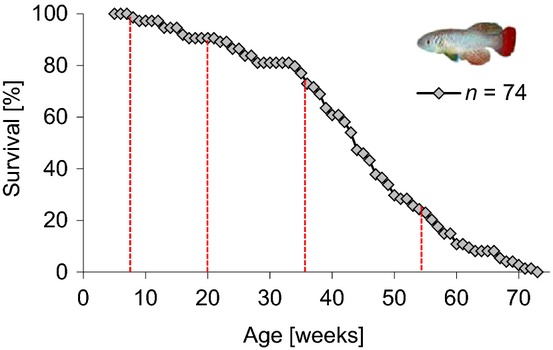
Survival curve of *Nothobranchius furzeri* strain MZM-0703. Mean lifespan of that wild-derived strain (*n* = 74; housed in single tanks) was 43.5 weeks and maximum lifespan (10% survival) was 60 weeks. Based on the survival curve four age groups (8 weeks, 20 weeks, 36 weeks and 54 weeks) were determined for regeneration experiments.

To study the influence of aging on the regenerative potential, we analysed caudal fin regeneration at different ages of the wild-derived *Nothobranchius furzeri* strain MZM-0703. A lifespan experiment of single-housed male animals (*n* = 74) revealed a mean lifespan of 43.5 weeks and a maximum lifespan (10% survival) of 60 weeks (Fig.1). Based on the survival curve, we decided to study fin regeneration in four different age groups. The young age group consisted of 8-week-old fish that can be considered as young adults, because the animals reach sexual maturity at an age of 3–5 weeks. Ninety-nine per cent of the population was alive at that age. Middle-aged fish had an age of 20 weeks when 90% of the population was alive. Old fish had an age of 36 weeks (73% of the population was alive) but did not represent the most long-lived individuals. Instead, this age rather marks the beginning of increased mortality (Fig.1). The group of very old fish were 50–60 weeks old with a mean age of 54 weeks representing the oldest 24% of the population.

**Fig 2:**
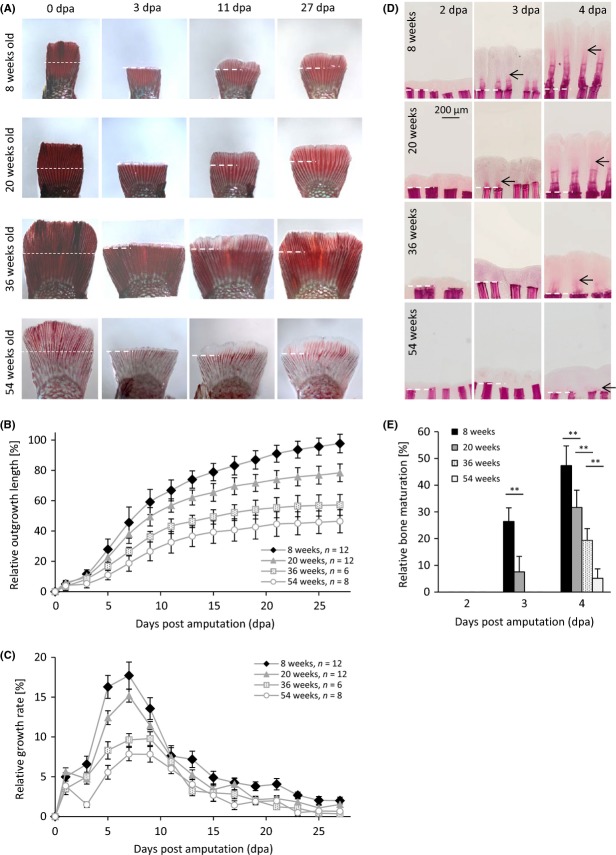
Outgrowth kinetic and bone maturation. (A) Examples of uninjured (0 days post amputation (dpa)) and regenerating caudal fins (3, 11, 27 dpa) from 8-, 20-, 36-, and 54-week-old fish. The white dashed line represents amputation plane. (B) The regeneration process of individual fish (*n* = 6–12 per age group) was monitored by taking pictures every second day. The length of outgrowth was related to the original fin size and given in percentage. Differences between all age groups were significant from 9 dpa onwards (Tukey’s HSD test at 9 dpa: 8 vs. 20 weeks, *P* < 0.05; 20 weeks vs. 36 weeks, *P* < 0.01; 36 vs. 54 weeks, *P* < 0.05). (C) The relative growth rate was determined by the increase in length between two time points. (D) Bones of the fin rays were stained with Alizarin Red at 2, 3 and 4 dpa in all age groups. Bone formation in the regenerating tissue was detectable at 3 dpa in the 8- and 20-week-old fish and at 4 dpa in all age groups (black arrows). White dashed line represents amputation plane. (E) Length of newly formed bones was measured and related to outgrowth length in 4–6 rays of 4–5 animals per time point and age group. Significant differences between age groups were detected using one-way ANOVA followed by Tukey’s HSD test (**P < 0.01).

As known for other teleost fish species, *N. furzeri* has the potential to completely regenerate its fins. We assessed regeneration of the caudal fin by determining the relative length of fin outgrowth every second day after amputation (Fig.2A). Each age group (8 weeks, 20 weeks, 36 weeks and 54 weeks) consisted of six to twelve male MZM-0703 fish. Animals from all age groups had the potential to regenerate their caudal fins, however, to a different degree (Fig.2B). The 8-week-old fish were able to nearly completely (98%) regenerate their amputated caudal fins within 27 days, whereas the 20-week-old fish only reached 78% of their original fin size. The ability to fully regenerate further declined in the 36-week-old fish that reached an average size of 57%. Strikingly, very old fish with a mean age of 54 weeks were only able to regenerate 46% compared to their original fin size. In addition, the majority of very old fish (five of eight fish) did not uniformly regenerate across the entire fin with some parts not showing outgrowth at all (Fig. S1A). This phenomenon of partial regeneration was only observed in the very old fish and not in any other age group. The difference in the relative length of outgrowth between young and very old fish was already significant at 3 days post amputation (dpa) and increased with time (Fig.2B). From 9 dpa onwards, the difference in outgrowth was significant between all age groups.

Because temperature is one of the important environmental factors influencing zebrafish fin regeneration (Boominathan & Ferreira, 2012), we wanted to know whether this holds also true for *N. furzeri*. Keeping 8-week-old fish at 33 °C compared to 27 °C resulted in an increased outgrowth at 3, 5 and 7 dpa (Fig. S1B). However, final outgrowth length was temperature independent, suggesting that temperature does not influence overall regenerative capacity.

We also determined the relative growth rate as the increase in outgrowth length between two time points. Young fish had significantly higher growth rates at 3, 5, 7 and 9 dpa than the very old fish (Fig.2C). The relative growth rates of the different age groups followed a similar pattern with the exception that the maximum growth rate of the two young age groups peaked at 7 days post amputation (8-week-old fish: 17.7% at 7 dpa, 20-week-old fish: 15.2% at 7 dpa). However, the two older age groups had similar growth rates at 7 and 9 dpa (36-week-old fish: 9.6% at 7 dpa and 9.8% at 9 dpa, 54-week-old fish: 7.8% at 7 dpa and 7.8% at 9 dpa). Overall, the growth rate pattern of the aged groups indicated a delayed regenerative process. Turquoise killifish continue to grow throughout their life under optimal conditions, although body growth usually declines with increasing age. We noted that the 8-week-old fish had a higher overall growth rate than the old fish (Fig. S2A). Therefore, we recalculated the length of outgrowth by taking the body growth into consideration. Even though relative outgrowth length changed, the difference between the age groups remained significant (Fig. S2B).

Because of the early difference in outgrowth kinetics (from 3 dpa onwards), we hypothesized that early events are crucial for the age-related differences in regenerative potential. We therefore focused on early time points and performed Alizarin Red staining at the second, third and fourth day post amputation to visualize bone formation (Fig.2D). There were no newly formed bones visible at 2 dpa in any of the age groups. At 3 dpa, newly formed bones were visible in the 8-week-old and 20-week-old fish, but not in older age groups. Interestingly, 54-week-old fish showed a strong delay in bone formation with only 5% mineralization at 4 dpa, at which we detected 10-fold higher values for 8-week-old fish (Fig.2E). Differences in bone formation were significant between all age groups.

**Fig 3:**
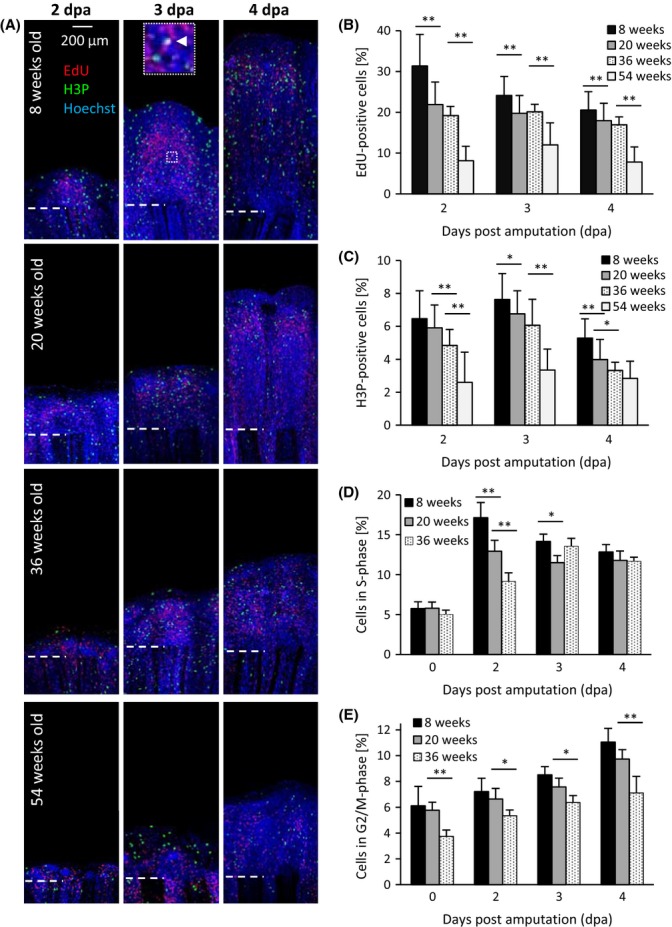
Number of proliferating cells is incre-ased in the regenerating fin of young fish compared to old fish. (A) The proliferation marker EdU (red) and H3P (green) as well as the cell nuclei (blue) were stained in optical sections of regenerating fins (2, 3 and 4 dpa) from all age groups. Cells with EdU and H3P double-labelling were only found in the young fish at 3 and 4 dpa (white arrow). White dashed line represents amputation plane. (B+C) Number of EdU- and H3P-positive cells was counted and related to total amount of cell nuclei. Young fish had significantly higher numbers of EdU- and H3P-positive cells than middle-aged and old fish. (D+E) Cells were isolated from uninjured (0 dpa) and regenerating tissue (2, 3 and 4 dpa) and sorted according to their DNA content. Number of cells with partially replicated DNA (S phase) and duplicated DNA (G2/M phase) was related to total cell number. Significant differences between age groups were detected using one-way ANOVA followed by Tukey’s HSD test (*P < 0.05, **P < 0.01).

We were further seeking insights into the proliferative state of the cells in the regenerating tissue. For that, we intraperitoneally injected EdU, which is incorporated into DNA during S phase, 30 min prior fixation. At 2 and 3 dpa, EdU-positive cell nuclei were mainly found in the proximal blastema region (Fig.3A). Towards 4 dpa, EdU-positive cells were also observed along the epidermal region of each fin ray in the 8-week- and 20-week-old fish. For quantification of EdU-positive cell nuclei, we analysed five sections per fin ray and used four to six fin rays per animal. Three to five animals were used for each time point and age group (Fig.3B). We observed the highest percentage of EdU-positive cells (31%) at 2 dpa in the 8-week-old fish. This number decreased to 24% at 3 dpa and to 21% at 4 dpa. A similar distribution was observed for the other age groups, except that both old age groups had their maximum percentage of EdU-positive cells at 3 dpa. At all time points, the 8-week-old fish showed a significant higher amount of EdU-positive cells than any other age group. Furthermore, we analysed phosphorylation of serine 10 in histone H3 (H3P), which serves as a marker of chromosome condensation during mitosis (Nechiporuk & Keating, 2002). H3P staining of regenerating fins in *N. furzeri* revealed a similar pattern as the EdU staining (Fig.3A). At all time points, 8-week-old fish had the highest percentage of H3P-positive cell nuclei, followed by 20-week-old fish, 36-week-old fish and 54-week-old fish (Fig.3C). At each time point, the difference between youngest and oldest fish was significant. Interestingly, an overlay of EdU- and H3P-positive cells indicating a G2 phase of less than 30 min was only found at 3 and 4 dpa in the 8-week-old-fish (Fig.3A, inset).

**Fig 4:**
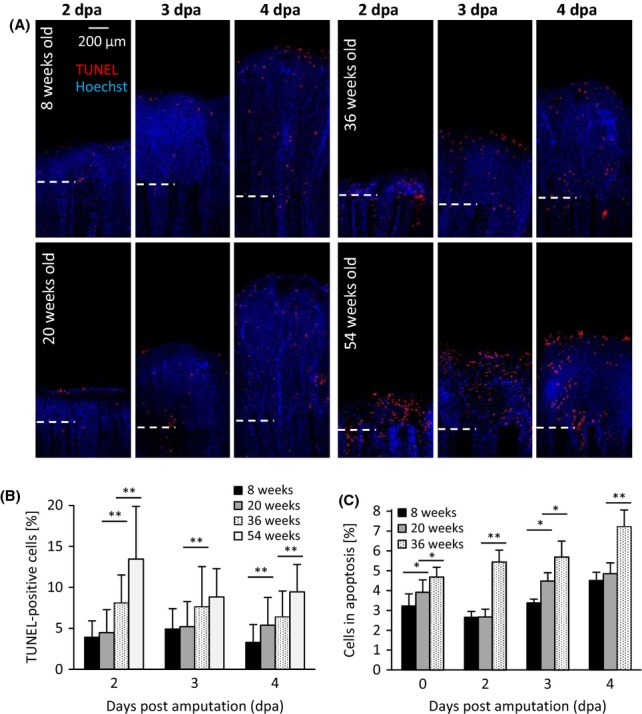
Number of apoptotic cells increased in the regenerating fin of old fish compared to young fish. (A) TUNEL staining (red) marked apoptotic cells in optical sections of regenerating fins (2, 3 and 4 dpa) from all age groups. White dashed line represents amputation plane. (B) Number of apoptotic cells was counted and related to total amount of cell nuclei. Old fish had significant higher numbers of apoptotic cells than young fish at all time points. (C) Cells were isolated from uninjured (0 dpa) and regenerating tissue (2, 3 and 4 dpa) and sorted according to their DNA content. Number of cells with fragmented DNA (apoptotic) was related to total cell number. Old fish had significantly higher numbers of apoptotic cells than young fish. Significant differences between age groups were detected using one-way ANOVA followed by Tukey’s HSD test (*P < 0.05, **P < 0.01).

To measure DNA content of newly formed cells and to determine the fraction of cells in S phase and G2/M phase, we used FACS analysis of 8-, 20- and 36-week-old fish (Fig.3D, E). At all time points and in all age groups, the percentage of cells in S phase was higher than that in the nonregenerating fins (0 dpa). In accordance with the EdU staining, the highest percentage of cells in S phase was observed at 2 dpa for the 8-week- and 20-week-old fish and at 3 dpa for the 36-week-old fish (Fig.3D). The highest percentage of cells in G2/M phase was found at 4 dpa for all age groups with 11.1% for the 8-week-old fish, 9.7% for the 20-week-old fish and 6.4% for the 36-week-old fish (Fig.3E). Next, we asked whether there are differences in the degree of apoptotic cells between the age groups during regeneration. TUNEL staining revealed that very old fish (54-week-old) had the highest proportion of apoptotic cells at all time points (Fig.4A,B). Determining the amount of apoptotic cells via DNA content analysis revealed a similar pattern as the TUNEL staining. At all time points, most apoptotic cells were present in the oldest (here: 36-week-old) fish (Fig.4C).

**Fig 5:**
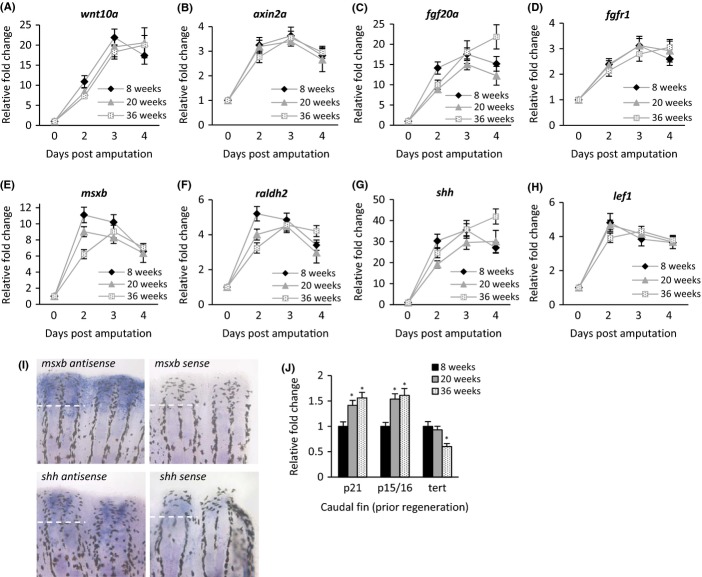
Expression levels of genes involved in fin regeneration are upregulated upon amputation in all age groups. (A–H) Quantitative RT–PCR was performed to determine gene expression in the regenerating fin (2, 3 and 4 dpa) of all age groups. Three samples per time point and age group were analysed consisting of 5–6 animals each. Gene expression was normalized to uninjured fin tissue (0 dpa) and to the reference genes *insr* and *ef-1*. Analysis of peak expression revealed that *raldh2*, *lef1*, *msxb* showed maximum expression at 2 dpa in the 8-week-old fish, while maximum expression was a day later (3 dpa) in the 36-week-old fish. In addition, *shh*, *fgf20a*, *fgfr1*, *wnt10a* also showed peak expression in the young fish at 3 dpa while in the old fish at 4 dpa. Earlier maximum expression of young fish was significant when plotting the relative expression difference between two time points into new graphs and comparing the slope of the best-fitted line using the binomial test (*P* = 0.0045). (I) Localization of *msxb* and *shh* expression was analysed by *in situ* hybridization with antisense probes. Both genes were highly expressed in the regenerating tissue. Sense probes served as negative controls. White dashed line represents amputation plane. (J) The cell-cycle inhibitor genes *p21* and *p15/p16* are higher expressed in uninjured fin tissue of aged fish compared to young fish, while the telomerase gene *tert* is less expressed in old fish (relative expression software tool, *P < 0.05).

We also wanted to know whether we could detect age-dependent differences in gene expression within the regenerating tissue. We selected eight genes that have been shown to be involved in fin regeneration of zebrafish and identified the orthologous genes in *N. furzeri* using the recently established transcript catalogue (Petzold *et al*., 2013). Selected genes included *wnt10a*, *axin2a*, *fibroblast growth factor 20a (fgf20a)*, *fibroblast growth factor receptor 1 (fgf1)*, *muscle segment homeobox b (msxb)*, *retinaldehyde dehydrogenase 2 (raldh2)*, *sonic hedgehog (shh)* and *lymphoid enhancer-binding factor 1 (lef1)*. Expression of all genes was significantly increased in the regenerating fin at 2, 3 and 4 dpa compared to nonregenerating fin (0 dpa) among all age groups (Fig.5A–H). Highest elevated expression levels were detected for *shh*, *wnt10a*, *msxb* and *fgf20a* (relative fold change ranged from 6- to 40-fold), while moderately elevated expression levels were observed for *raldh2*, *lef1*, *fgf1r* and *axin2a* (relative fold change ranged from two- to fivefold). Enhanced expression of *msxb* and *shh* was specific to regenerating fin tissue as shown by *in situ* hybridization (Fig.5I). When comparing peak expression within the same age group, four genes (*shh*, *fgf20a*, *fgfr1* and *wnt10a*) had their expression maximum at 3 dpa in the 8-week-old fish, while the expression of the same genes in the 36-week-old fish peaked one day later, at 4 dpa. Similarly, three genes (*raldh2*, *lef1* and *msxb*) showed maximum expression at 2 dpa in the 8-week-old fish and at 3 dpa in the 36-week-old fish. Statistical analysis revealed that for six of eight genes, expression in 8-week-old fish peaked earlier than in 20-week-old fish and finally followed by 36-week-old fish. This suggests that older fish show a delay in gene expression already at an early stage during regeneration. Finally, we measured the expression of the cell-cycle inhibitor genes *p21* and *p15/p16* in uninjured caudal fin tissues and found higher expression levels of both genes in older animals. On the other hand, the telomerase gene *tert* was expressed to a lower degree in the uninjured fin of old fish (Fig.5J).

## Discussion

The regenerative capacity is higher at early develomental stages than at adult stages. To investigate whether the regenerative capacity declines during adulthood, we used the short-lived killifish *Nothobranchius furzeri*. Like other teleost fishes, *N. furzeri* is able to regenerate its caudal fin after amputation. Due to its exceptional short lifespan, this killifish species is very suitable to study the impact of aging on regenerative processes. Our data show that there is a significant decline in the regenerative capacity of the fin with aging. Young fish regenerated more rapidly and almost completely restored their original fin size in comparison with older fish. This observation is surprising, because it has been recently suggested that aging does not affect regenerative processes in animals with strong regenerative capabilites such as salamanders and zebrafish (Seifert & Voss, 2013; Sousounis *et al*., 2014). In fact, there are several reports with opposing results. In newts, for example, the entire eye lens can regenerate and neither repeated regeneration nor old age altered the regenerative capacity of the lens (Eguchi *et al*., 2011). In zebrafish, two studies reported that old animals show impaired fin regeneration and that the time to regenerate 50% of the fin increased with advanced age (Tsai *et al*., 2007; Anchelin *et al*., 2011). One survey found slight age-dependent differences in fin outgrowth at late regeneration stages (Shao *et al*., 2011), while another study stated no decline in the capacity to regenerate heart and fin of aged zebrafish at all (Itou *et al*., 2012). One explanation for these discrepancies could be the diverging classification of age groups as in some of the studies zebrafish with an age of 18–24 months were classified as old animals. Considering a mean lifespan of 42 months for zebrafish, with the longest living individual surviving 66 months (Gerhard *et al*., 2002), old animals in some of the studies might represent rather middle-aged zebrafish. We studied regeneration kinetics in *N. furzeri* in four distinct age groups and noticed a continous decline in regeneration capacity with age, with young fish almost completely restoring their original fin size, while very old fish only reaching 46%.

It has been hypothesized that one reason for the high regenerative capabilities of fish is because fish continue to grow throughout their adult life (Poss, 2010). This is also true for *N. furzeri*, where young adult fish still show considerable body growth, but growth rates decrease with advancing age. Furthermore, the number of proliferating cells, which are important for regeneration, should be higher in tissues that still show increased growth rates. To test this hypothesis, we measured the proportion of proliferating cells in fin tissue before and during regeneration in the four age groups. Using different methods, our data show that the number of proliferating cells during regeneration is significantly higher in young fish than in old fish. Our results also indicate, although to a lesser extent, that the fin tissue of old fish already contains lower numbers of proliferating cells before regeneration compared to young fish. Furthermore, through double-staining with EdU and H3P, we observed that the cell cycle is accelerated particularly in the regenerating fin of young fish. This is in agreement with observations in zebrafish that show the cell cycle is accelerated during fin regeneration (Nechiporuk & Keating, 2002). Thus, our results suggest that age-related differences in the regenerative capacity result from higher numbers of proliferating cells and increased cell division rates in young animals.

Before and during regeneration, the proportion of apoptotic cells is increased in old fish compared with young fish. Deregulation of apoptosis has been frequently associated with the aging process; however, it is still debatable whether aging suppresses or enhances apoptosis *in vivo*. Recently, it has been documented that the occurrence of apoptosis within 24 h upon amputation is crucial to initiate fin regeneration in zebrafish (Gauron *et al*., 2013). Our data support significant changes in the number of apoptotic cells during regeneration between young and old fish but not at different time points within the same age group; however, we only analysed later time points during regeneration than 24 h.

A further explanation for the observed age-related differences in regeneration could be that tissues of young fish have easier access to embryonic programmes than aged tissues, which have been largely quiescent for longer time (Poss, 2010). The Wnt, Fgf and hedgehog signalling pathways, for example, play crucial roles during embryonic development and have been identified to be highly upregulated during zebrafish fin regeneration (reviewed in Tal *et al*., 2010). We determined expression levels of genes that are involved in those pathways such as *wnt10a, axin2a* and *lef1* in Wnt signalling; *fgf20a* and *fgfr1* in Fgf signalling; and *shh* in hedgehog signalling. All genes were significantly upregulated during fin regeneration in *N. furzeri*. For example, *axin2a* was upregulated by the factor 2.5–3.5 and *lef1* was upregulated by factor 3.8–4.9, which is in good agreement with zebrafish microarray data (Schebesta *et al*., 2006). An interesting observation is that when determining the maximum fold change in gene expression during regeneration, six of eight genes were highest upregulated at an earlier time point in young fish than in old fish. This suggests a more rapid initiation and progress of the regeneration process in young fish. An age-related increase in senescent cells could also contribute to this observation. For instance, it has been shown that the expression of the cell-cycle inhibitors *p21* and *p15/p16*, which are considered as markers for senescence, is increased in aged fish (Graf *et al*., 2013). We could observe that both *p21* and *p15/p16* are expressed at higher levels in the uninjured fin of old fish, providing another explanation for the delayed initiation and progression of the regeneration process in *N. furzeri*.

Recently, dysfunction and exhaustion of stem cells has been considered as a hallmark of aging (Lopez-Otin *et al*., 2013). Regeneration of muscle tissue, for example, depends on satellite cells (muscle stem cells) whose function has been shown to decline with age in mice (Conboy *et al*., 2005). A recent study suggests that these cells start to proliferate in young mice after injury but enter an accelerated state of senescence in old mice (Sousa-Victor *et al*., 2014). Interstingly, it has been suggested that fin regeneration in fish and limb regeneration in salamanders do not seem to require multipotent stem cells but rather involve dedifferentiation of existing cells (Sandoval-Guzman *et al*., 2014). This mechanism of dedifferentiation might be responsible for the higher regenerative capacity of fish and amphibians in comparison with mammals. Lineage tracing experiments in zebrafish fin and salamander limb regeneration have shown that cells, which form the blastema and then grow out to replace the missing portion of the fin or limb, keep a memory of their tissue origin (Kragl *et al*., 2009; Tu & Johnson, 2011). However, these experiments cannot answer the question whether blastema formation occurs via dedifferentiation or via the activation of resident tissue stem cells. It is likely that both processes contribute to regeneration to differing degrees in various tissues (Tanaka & Reddien, 2011). Thus, it is tempting to speculate that the exhaustion of tissue-specific progenitor/stem cells are responsible for the age-related difference in *N. furzeri* fin regeneration. In future, it will be interesting to gain further insights into this fundamental relationship between aging and regeneration by studying which cells are most affected during aging and how age-dependent factors regulate regenerative capacity. A better understanding of the age dependency of regenerative processes may help to test and translate new discoveries into effective applications in regenerative medicine.

## Experimental procedures

### Fish maintenance

Turquoise killifish (*Nothobranchius furzeri*) of the strain MZM-0703 were collected in south-western Mozambique in 2007 and represent a wild-derived *N. furzeri* strain. The animals were raised in groups of 12–14 fish per 40-litre tank at 26 ± 1 °C under a light:dark regime of 12:12 h. Fry and young fish until the age of 6 weeks were fed on brine shrimp (*Artemia*) twice a day, while young and adult fish starting from an age of 3 weeks were fed on red mosquito larvae (Chironomidae) once a day. For lifespan and regeneration experiments, male fish were kept in single housing (two separated fish per 5-litre tank) at 27 °C in the stand-alone unit V30 (Aqua Schwarz, Göttingen, Germany) or in seperated tanks at 33 °C. All animal experiments were performed according to the ‘Principles of laboratory animal care’ as well as to the current version of the German Law on the Protection of Animals.

### Age groups

Initially, fin regeneration was analysed among three age groups (young: 8-week-old, middle-aged: 20-week-old and old: 36-week-old). In subsequent experiments, fin regeneration was analysed in very old fish (54-week-old) and in young (8-week-old) and middle-aged (20-week-old) fish for comparison. There was no significant difference between initial experiments and subsequent experiments among young and middle-aged fish; hence, results include data from both experiments. Due to the limited number of very old fish (54-week-old), some aspects of regeneration were only analysed in young, middle-aged and old fish.

### Fin regeneration assay

Six to twelve animals at the age of 8 weeks, 20 weeks, 36 weeks and 54 weeks were anesthetized with Tricaine/MS-222 (0.2 mg mL^−1^; Sigma-Aldrich, Munich, Germany), and 50% of the caudal fin was amputated with a scalpel. Fins of individual fish were photographed using the stereo microscope Lumar V12 (Zeiss, Jena, Germany) directly after amputation and every second day post amputation including the first day for the duration of 27 days. Outgrowth length of concentric fin rays was determined for every time point in pixels and converted into millimetre using Photoshop (Adobe, San Jose, CA, USA). Next, the so-determined outgrowth length was related to the original length of the amputated fin. The regenerative growth rate was determined by the increase in outgrowth length between two time points in relation to the length of the original amputated fin. Significant differences between age groups were analysed at each time point using one-way ANOVA followed by pairwise comparison using Tukey’s HSD test.

### Histological staining and immunohistochemistry

Histological analyses of regenerating fin tissue were carried out at 2, 3 and 4 dpa. Fin tissue was fixed in 4% paraformaldehyde/PBS overnight at 4 °C and stored in methanol at −20 °C. For visualization of mature bones, fin tissue was rehydrated stepwise through a methanol series and stained with 0.5% KOH containing 0.5% Alizarin Red S (Sigma-Aldrich, Munich, Germany) for 2 h as previously described for zebrafish (Sire *et al*., 1997). Digital mosaic images were acquired in bright field at 10× magnification with the virtual microscope VS120-S1 (Olympus, Hamburg, Germany). Labelling of proliferative cells was achieved by intraperitoneal injections of 10 mM 5-ethynyl-2′-deoxyuridine (EdU) solution (10 μL g^−1^ body weight) at 30 min prior fixation in 4% paraformaldehyde/PBS and storage in methanol at −20 °C. Fins were rehydrated stepwise with PBSTx (0.3% Triton X-100 in 1x PBS), permeabilized with ice-cold acetone (−20 °C) for 20 min and rinsed twice with ddH_2_O. Afterwards, fins were digested with 1 mg mL^−1^ collagenase/dispase (Roche, Mannheim, Germany) for 45 min at room temperature and refixed with 4% paraformaldehyde/PBS for 15 min. After bleaching with 1.5% H_2_O_2_ and 1% KOH for 20 min, the Click-iT® EdU AlexaFluor® 594 Imaging Kit (Life Technologies, Darmstadt, Germany) was used to visualize incorporated EdU. For the detection of apoptotic cells, fins were harvested and fixed in 4% paraformaldehyde/PBS as described above. TUNEL staining was performed by incorporation of biotinylated dUTP using terminal deoxynucleotidyl transferase according to the instructions of the manufacturer (Roche). Biotinylated dUTP was localized by CY3-conjugated streptavidin (Life Technologies). Fin samples treated with EdU were costained with a primary H3P antibody to detect mitotic cells. Therefore, fins were washed with 3% BSA/PBS, and unspecific binding sites were blocked with a PBSTx buffer containing 20% normal goat serum and 5% BSA. The polyclonal antiphospho histone H3 antibody #06-570 (Merck-Millipore, Schwalbach, Germany) was diluted 1:200 in a PBSTx buffer containing 10% normal goat serum and 1% BSA and was applied overnight at 4 °C. Hereafter, fins were washed three times in PBSTx buffer and incubated with the secondary antibody (Alexa Fluor 488 goat anti-rabbit IgG or streptavidin-Cy3, 1:200; Life Technologies) in PBSTx buffer containing 10% normal goat serum, 1% BSA and 0.01% Hoechst overnight at 4 °C. Prior to embedding in VECTASHIELD® Mounting Medium (Vector Laboratories, Burlingame, CA, USA), fins were washed three times in PBS. Optical mosaic section of the fluorescence signals was acquired at 10× magnification with a width of 1.5 μm using the Axio Imager Z1 microscope (Zeiss, Jena, Germany). Automated cell counting was performed with the open-source software CellProfiler (Kamentsky *et al*., 2011). Cell nuclei in the regenerating outgrowth that were stained with the respective antibody were related to the total number of nuclei as detected by Hoechst. The mean cell number of five adjacent central optical sections was calculated for one fin ray. Four to six regenerating fin rays were used from 3 to 5 animals of each age group and time point. Significant differences between age groups were analysed at each time point using ANOVA followed by pairwise comparison using Tukey’s HSD test.

### FACS analysis

Fin tissue was dissociated by vigorous shaking in 1 mg mL^−1^ collagenase/dispase solution (Roche) for 20 min at 37 °C. Collagenase was inactivated with 20% FBS/DMEM, and the suspension was passed through a 40-μm mesh. The suspension was centrifuged for 10 min, and the cell pellet was resuspended and incubated in 2% paraformaldehyde/PBS for 10 min at room temperature. Afterwards, cells were precooled for 1 min on ice and then permeabilized with ice-cold methanol, which was added dropwise while shaking. After incubation for 30 min on ice, the cell suspension was washed with PBS and stained with 0.05% Hoechst/PBS. After a final wash step with PBS, cells were analysed with the flow cytometer FACSCanto II (BD Biosciences, Heidelberg, Germany) using forward and side scatter parameters to exclude cell debris. Five to six animals were used for analysis per time point and age group, while 10–14 animals were used for analysis of uninjured fin tissue. Data were analysed with FLOWING Software (version 2.5.0, www.flowingsoftware.com). Significant differences between age groups were analysed at each time point using ANOVA followed by pairwise comparison using Tukey’s HSD test.

### *In situ* hybridization

Whole mount *in situ* hybridization of regenerating fins (3 dpa) was performed as previously described (Perner *et al*., 2007). Fins were hybridized with digoxigenin-labelled RNA probes consisting of the sense or antisense sequence of either the *N. furzeri*-specific *shh* or *msxb* sequence (see Table S1 for primer sequences). The probes were detected using anti-digoxigenin-AP Fab fragments (Roche) and stained with NBT/BCIP-solution (Roche). Stained whole mounts were cleared with clearing solution (1/3 benzoyl-alcohol and 2/3 benzoyl-benzoate) and photographed using a stereomicroscope (SteREO Discovery.V8, Zeiss).

### Gene expression analysis

Amputated fin tissue was used for normalization of gene expression and referred to as 0 dpa. Fin outgrowth at 2, 3 and 4 dpa was re-amputated at the original amputation plane, and fins were pooled from 5 to 6 animals resulting in three tissue samples per time point and age group. All tissue samples were snap-frozen on dry ice and stored at −80 °C. Total RNA was isolated using the RNeasy Micro kit including DNase digestion as suggested by the manufacturer (Qiagen, Hilden, Germany). Reverse transcription of RNA was carried out with the iScript cDNA Synthesis Kit according to the manufacturer’s manual (Bio-Rad, Munich, Germany). Negative controls without reverse transcriptase and without RNA were included in each cDNA synthesis. Quantitative PCR was performed with the SYBR® GreenER™ qPCR SuperMix (Life Technologies) using the CFX384 Touch™ Real-Time PCR Detection System (Bio-Rad, Munich, Germany). Each reaction consisted of 3.5 μL qPCR SuperMix, 0.15 μL forward and reverse primer (100 pmol μL^−1^) each and 3.2 μl cDNA template (1:10 dilution). Cycling conditions were as suggested by the qPCR SuperMix manual except for an annealing and elongation temperature at 58 °C instead of 60 °C. Each template was measured in technical triplicates, and PCR efficiency was between 95 and 100%. Primer sequences for the different genes are given in Table S1 (Supporting information). The *insulin receptor (insr)* and *elongation factor 1 (ef-1)* genes were used as reference. Gene expression was analysed using the relative expression software tool (REST) which is based on the ΔΔC_t_-method (Pfaffl *et al*., 2002). For further statistical evaluation of maximum expression, relative expression differences from time point to time point (0→2, 2→3, 3→4 dpa) of each gene and age group were plotted and the lines of best fit were determined. A steeper slope and an earlier crossing of the *x*-axis of these lines indicate an earlier expression peak, so these values were compared for statistical differences using the binomial test.

## Supporting Information

**Fig. S1** Regenerative impairment in very old fish and temperature effect on regeneration. **Fig. S2** Regenerative length measurements including body growth.

**Table S1** Primer list.
