# Supplementary material for: Age-dependent decline in fin regenerative capacity in the short-lived fish Nothobranchius furzeri
Source: Aging Cell. 2015 Jun 29;14(5):857–66. doi: 10.1111/acel.12367 (PMC4568973; doi:10.1111/acel.12367)
Supplement: Supplementary file 1 [file acel0014-0857-sd1.pdf]

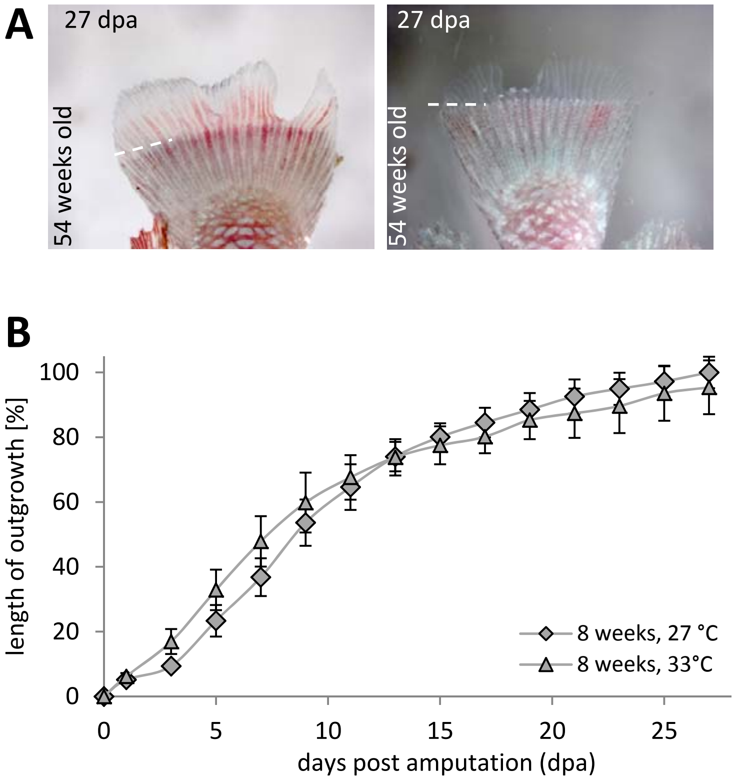

Figure S1:

Regenerative impairment in very old fish and temperature effect on regeneration. (**A**) Two examples of impaired fin regeneration in very old fish after 27 dpa. (**B**) Higher temperature (33°C) results in a more rapid outgrowth at 3, 5, and 7 dpa (one-way ANOVA, $p<0.05$).

Supplementary Figure 1

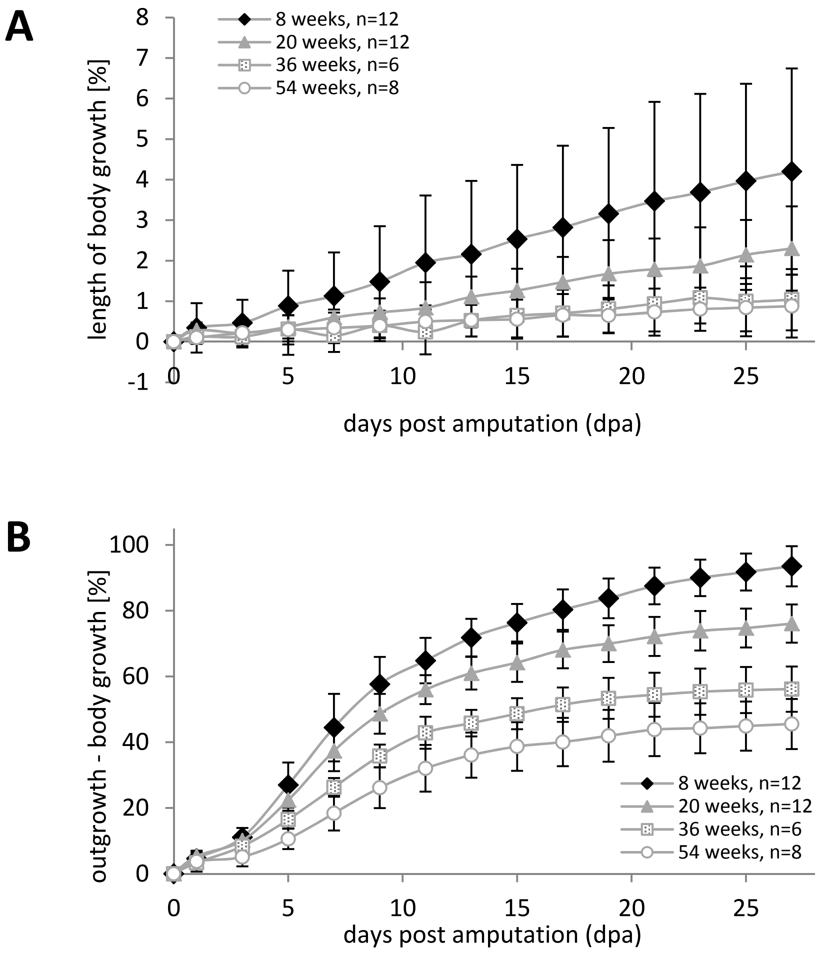

Figure S2: Regenerative length measurements including body growth. (**A**) Young fish show higher body growth than old fish. (**B**) Relative regenerative length subtracted by relative body growth at each time point.

Supplementary Figure 2
