# Supplementary material for: Age-dependent decline in fin regenerative capacity in the short-lived fish Nothobranchius furzeri
Source: Aging Cell. 2015 Jun 29;14(5):857–66. doi: 10.1111/acel.12367 (PMC4568973; doi:10.1111/acel.12367)
Supplement: Supplementary file 2 [file acel0014-0857-sd2.docx]

**Table S1 Primer list**

| **Primer** | **Forward sequence** | **Reverse sequence** |
| --- | --- | --- |
| axin2a | CCTCACCTTGGGTCACTTCA | TCAGTCCATCCTCTCCACCT |
| fgf20a | GGACGAGGAAGGACCACAG | CTGTTCCCTGAAGACGCATT |
| fgfr1 | GGAACGTCCTGGTTACAGAAAG | GGTCAAAAAGTGCCTCTGGA |
| insr | TGCCTCTTCAAACCCTGAGT | AGGATGGCGATCTTATCACG |
| lef1 | TCCTCTCTCCTCCTGTTGCT | GTGGGCTCGTTAGGATTCAC |
| 1.msxb | CGCCTTTTCAGTGGGAGA | GGGCTTTCTGTTGGTCTTGT |
| 2.msxb | GCCCAAAGACATGACAACAGAGAAAGGATA | GACAAATAATACATCCCATAAGTGACCGGTC |
| raldh2 | GTCCACAGATCAGTCGGGA | CACGCTGGAGAAAACCTTG |
| 1.shh | GAGCAAATACGGGACTCTGTC | GGTCTCTCACCGCCTTCTT |
| 2.shh | TCCGAGGAGTCCCTCCACTACGA | GTCCAACAACCATGTCCCCAGCTG |
| wnt10a | TCGTCAACTCCCAGAACAAG | CTACAGACACATCGGGCTCA |
